# Association of ambulatory blood pressure with coronary microvascular and cardiac dysfunction in asymptomatic type 2 diabetes

**DOI:** 10.1186/s12933-022-01528-2

**Published:** 2022-05-28

**Authors:** Jian L. Yeo, Gaurav S. Gulsin, Emer M. Brady, Abhishek Dattani, Joanna M. Bilak, Anna-Marie Marsh, Manjit Sian, Lavanya Athithan, Kelly S. Parke, Joanne Wormleighton, Matthew P. M. Graham-Brown, Anvesha Singh, J. Ranjit Arnold, Claire Lawson, Melanie J. Davies, Hui Xue, Peter Kellman, Gerry P. McCann

**Affiliations:** 1 Department of Cardiovascular Sciences, University of Leicester and the National Institute for Health Research Leicester Biomedical Research Centre, Glenfield Hospital, Leicester, UK; 2 Diabetes Research Centre, University of Leicester and the NIHR Leicester Biomedical Research Centre, Leicester, UK; 3 Leicester Diabetes Centre, Leicester General Hospital, Leicester, UK; 4 Department of Health and Human Services, National Heart, Lung, and Blood Institute, National Institutes of Health, Bethesda, MD USA

**Keywords:** Blood pressure, Type 2 diabetes, Ambulatory blood pressure, Diabetic cardiomyopathy, Myocardial perfusion reserve

## Abstract

**Background:**

Type 2 diabetes (T2D) and hypertension commonly coexist and are associated with subclinical myocardial structural and functional changes. We sought to determine the association between blood pressure (BP) and left ventricular (LV) remodeling, systolic/diastolic function, and coronary microvascular function, among individuals with T2D without prevalent cardiovascular disease.

**Methods:**

Participants with T2D and age-, sex-, and ethnicity-matched controls underwent comprehensive cardiovascular phenotyping including fasting bloods, transthoracic echocardiography, cardiovascular magnetic resonance imaging with quantitative adenosine stress/rest perfusion, and office and 24-h ambulatory BP monitoring. Multivariable linear regression was performed to determine independent associations between BP and imaging markers of remodeling and function in T2D.

**Results:**

Individuals with T2D (n = 205, mean age 63 ± 7 years) and controls (n = 40, mean age 61 ± 8 years) were recruited. Mean 24-h systolic BP, but not office BP, was significantly greater among those with T2D compared to controls (128.8 ± 11.7 vs 123.0 ± 13.1 mmHg, p = 0.006). Those with T2D had concentric LV remodeling (mass/volume 0.91 ± 0.15 vs 0.82 ± 0.11 g/mL, p < 0.001), decreased myocardial perfusion reserve (2.82 ± 0.83 vs 3.18 ± 0.82, p = 0.020), systolic dysfunction (global longitudinal strain 16.0 ± 2.3 vs 17.2 ± 2.1%, p = 0.004) and diastolic dysfunction (E/e’ 9.30 ± 2.43 vs 8.47 ± 1.53, p = 0.044) compared to controls. In multivariable regression models adjusted for 14 clinical variables, mean 24-h systolic BP was independently associated with concentric LV remodeling (β = 0.165, p = 0.031), diastolic dysfunction (β = 0.273, p < 0.001) and myocardial perfusion reserve (β = − 0.218, p = 0.016). Mean 24-h diastolic BP was associated with LV concentric remodeling (β = 0.201, p = 0.016).

**Conclusion:**

24-h ambulatory systolic BP, but not office BP, is independently associated with cardiac remodeling, coronary microvascular dysfunction, and diastolic dysfunction among asymptomatic individuals with T2D. (Clinical trial registration. URL: https://clinicaltrials.gov/ct2/show/NCT03132129 Unique identifier: NCT03132129).

**Supplementary Information:**

The online version contains supplementary material available at 10.1186/s12933-022-01528-2.

## Background

Type 2 diabetes (T2D) and hypertension are associated with cardiac structural and functional alterations which predispose patients to a heightened risk of cardiovascular disease (CVD) and in particular, heart failure [1, 2]. The combined impact of T2D and hypertension on cardiac structure and function is a major driver for the additive risk of adverse cardiovascular outcomes and death (2–fourfold greater in patients where both conditions coexist) [3]. Cardiac imaging techniques can identify subtle abnormalities of cardiac structure and function before symptoms develop, described as subclinical cardiac dysfunction or stage B heart failure [4]. Abnormalities include concentric left ventricular (LV) remodeling, left atrial dilatation, diastolic dysfunction, and reduced global longitudinal strain, which have been associated with increased risk of cardiovascular events and heart failure over and above traditional risk factors [1, 5, 6].

Cardiovascular magnetic resonance imaging (MRI) is the gold standard for assessment of cardiac volumes, mass, and ejection fraction, and permits detailed evaluation of myocardial strain [7]. Additionally, adenosine stress perfusion imaging has the ability to provide accurate quantification of myocardial blood flow and perfusion reserve [8], which, in the absence of epicardial coronary disease, is indicative of microvascular dysfunction and strongly related to cardiovascular outcomes in people with diabetes [9]. No studies to date have assessed the associations between MRI-measured cardiovascular structure and function with office or ambulatory blood pressure (BP) in people with T2D.

Ambulatory measurement is the recommended method for assessment of BP. Ambulatory SBP, rather than office SBP, has been shown to be an independent predictor of the development or progression of peripheral arterial disease in people with T2D [10]. Furthermore, people with T2D who were older, had longer diabetes duration, and higher prevalence of cerebrovascular, peripheral artery disease, and microvascular complications had higher ambulatory SBP variability [11]. The use of ambulatory BP monitoring in high-risk coronary artery disease patients also helped improve BP control and influenced anti-hypertensive medication changes [12].

The aim of this study was to determine the associations between BP and cardiac remodeling, function, and myocardial perfusion reserve (MPR) in asymptomatic individuals with T2D and no prior history of CVD. We hypothesized that BP is an independently associated with imaging markers of myocardial dysfunction, and that ambulatory BP is more strongly associated to these markers than office BP.

## Methods

### Study population

In this single center, prospective observational study: Prevalence and determinants of subclinical cardiovascular dysfunction in adults with T2D (PREDICT, NCT 03132129), participants were recruited from primary care services in Leicestershire, UK. Participants were aged ≥ 18 to ≤ 75 years with a diagnosis of T2D and no prior history, signs, or symptoms of CVD (including symptomatic coronary, peripheral or cerebrovascular disease, valvular heart disease, arrhythmias, or heart failure). Exclusion criteria were diagnosis of Type 1 diabetes mellitus, estimated glomerular filtration rate < 30 mL/min/1.73 m^2^, or absolute contraindication to MRI. Participants with common co-morbidities associated with T2D such as obesity, treated hypertension, and mild dyspnea were included. Age-, sex- and ethnicity-matched healthy volunteers were enrolled for comparison. Ethical approval was provided by the UK Health Research Authority Research Ethics Committee (reference 17/WM/0192). All participants provided written informed consent.

### General examinations

Demographics, medical history, and anthropometric measurements were collected. Smoking status was recorded as never smoked, ex-smoker, or current smoker. Hypertension and hypercholesterolemia were determined by self-reporting by participants or prescribed medication to treat these conditions. A fasting blood sample was collected for biochemical profile including glycosylated hemoglobin (HbA1c), full blood count, lipid profile, liver function, renal function, and N-terminal pro B-type natriuretic peptide (NT-proBNP) and analyzed in an accredited National Health Service pathology lab at the University Hospitals of Leicester. Blood samples were collected on the same day as the imaging procedures.

### Blood pressure measurement

Office BP was measured using the Omron M6 (Hoofddorp, Netherlands) monitor in a seated position with an appropriately sized brachial cuff after 10 min of quiet rest, on the non-dominant arm. The average of three recordings were used for office BP.[13]. Ambulatory BP was measured over 24 h with a BP monitor (Space lab model 90207, Snoqualmie, Washington, USA) previously validated by the British Hypertension Society protocol [14]. Daytime BP readings were taken between 0700 to 2200 h, in 20-min intervals while night-time BP readings were taken between 2200 to 0700 h in 30-min intervals. Non-dipping pattern was defined as night-time to daytime systolic BP (SBP) ratio of > 0.9 [15]. The percentage of successful ambulatory BP recordings was recorded.

### Echocardiographic measurements

Transthoracic echocardiography was performed by one of two British Society of Echocardiography accredited operators (AMM or MS, each have at least 20 years of experience of performing and reporting transthoracic echocardiography) using an iE33b system with X5-1 transducer (Phillips Medical Systems, Best, Netherlands) to assess diastolic function. Images were acquired and reported as per the American Society of Echocardiography guidelines [16]. Early (E) and late (A) diastolic mitral inflow velocities, and early diastolic mitral annular velocities (e’) were assessed by Doppler echocardiography. Intra- and inter-observer variability for E/A and E/e’ was assessed in 10 participants, selected randomly for repeat image analysis. For intra-observer variability assessment, the duration between measurements was at least 2 weeks apart.

### Cardiovascular MRI

Cardiac MRI was performed using a standardized protocol on a 3-Tesla Siemens Skyra scanner (Erlangen, Germany) as previously described [17]. Perfusion images were acquired following vasodilator stress with adenosine (140–210 µg/kg/min) infusion for 3–5 min. Adequate hemodynamic response was determined by increased heart rate of 10%, 10 mmHg drop in systolic BP, and/or self-reported mild symptoms (e.g., chest tightness, tachypnoea, flushing). At peak stress, a gadolinium-based contrast agent (0.075 mmol/kg gadoteric acid, Dotarem) was injected followed by a 20 mL bolus of normal saline, at a rate of 5 mL/s. Perfusion images were acquired at three short-axis LV planes (basal, mid-ventricular, and apical). Rest imaging was performed approximately 10 min after stress. Quantitative myocardial blood flow analyses were performed using a dual-sequence gradient echo method with inline automated reconstruction and post-processing for myocardial blood flow quantification [18]. Contours were manually drawn if automated contours were incorrect. MPR was derived as the ratio of stress to rest blood flow. Pre- and post-contrast T1 mapping was performed using the Modified Look-Locker sequence. Late gadolinium enhancement images were acquired at least 5 min after rest perfusion for assessment of silent myocardial infarct and focal myocardial fibrosis.

### MRI image analysis and markers of cardiac dysfunction

Cardiac MRI images were analyzed using cvi42 (Version 5.10.1, Circle Cardiovascular Imaging, Calgary, Alberta, Canada) by an observer blinded to participant demographic and clinical details as previously described [17]. LV mass to end-diastolic volume ratio (LVM/V) was calculated as a marker of concentric remodeling. LV strain values are presented as absolute values, where lower values indicate worse myocardial mechanics, to avoid confusion with negative values which represent myocardial shortening [19].

Perfusion images were firstly assessed qualitatively (GPM) for regional perfusion defects indicative of ischemia due to epicardial coronary disease as per clinical standards [20, 21]. In order to assess coronary microvascular function, participants with regional ischemia typical of macrovascular epicardial disease were excluded from quantitative myocardial blood flow analysis. Late gadolinium enhancement (LGE) images were assessed qualitatively for scarring and individuals with LGE indicative of previous silent myocardial infarction were also excluded from perfusion analysis. Myocardial extra-cellular volume fraction (ECV), a surrogate marker of diffuse interstitial fibrosis, was calculated from pre- and post-contrast T1 maps [22].

### Key imaging outcome measures

In this study, we have focused on these key measures of subclinical cardiac dysfunction which have been shown to be abnormal in asymptomatic T2D versus controls include: LVM/V, MPR, LV global longitudinal strain (GLS), E/e’ [23] and myocardial ECV [24].

### Statistical analysis

Normality was assessed using histograms and Q-Q plots. Continuous data are expressed as mean ± standard deviation if normally distributed or median (interquartile range) if not. Groups were compared using independent-sample T-test or Mann–Whitney test as appropriate. Categorical data were reported as absolute values (percentages) and compared using chi-squared test. Intra- and interobserver variability for E/A and E/e’ ratios were assessed using intra-class correlation coefficient (ICC) including 95% confidence interval, using a two-way mixed, single measurement model with absolute agreement [25].

Univariable associations between BP and imaging outcome measures were assessed using Pearson correlation coefficients in participants with T2D. Multivariable linear regressions were performed to assess independent associations between office and ambulatory BP measurements and imaging measures of cardiac dysfunction (LVM/V, MPR, GLS, E/e’, and myocardial ECV). Each BP variable (office SBP and diastolic BP (DBP), ambulatory SBP and DBP over 24 h, during daytime and night-time) was added separately into a linear regression model adjusting for relevant clinical variables according to previous literature: age, male sex, white ethnicity, never smoked, body mass index (BMI), diabetes duration, HbA1c, estimated glomerular filtration rate, albuminuria, NT-proBNP, number of anti-hypertensive(s) prescribed, insulin, sodium-glucose co-transporter-2 inhibitor, and glucagon-like peptide-1 receptor agonist use which have BP reduction effect [26, 27]. Non-parametric continuous variables were logarithmic transformed before adding into the linear regression model. Statistical analysis was performed using Statistical Package for Social Sciences version 26.0 (SPSS Inc. Chicago, Illinois, USA). A p-value < 0.05 was considered statistically significant.

## Results

**Fig. 1:**
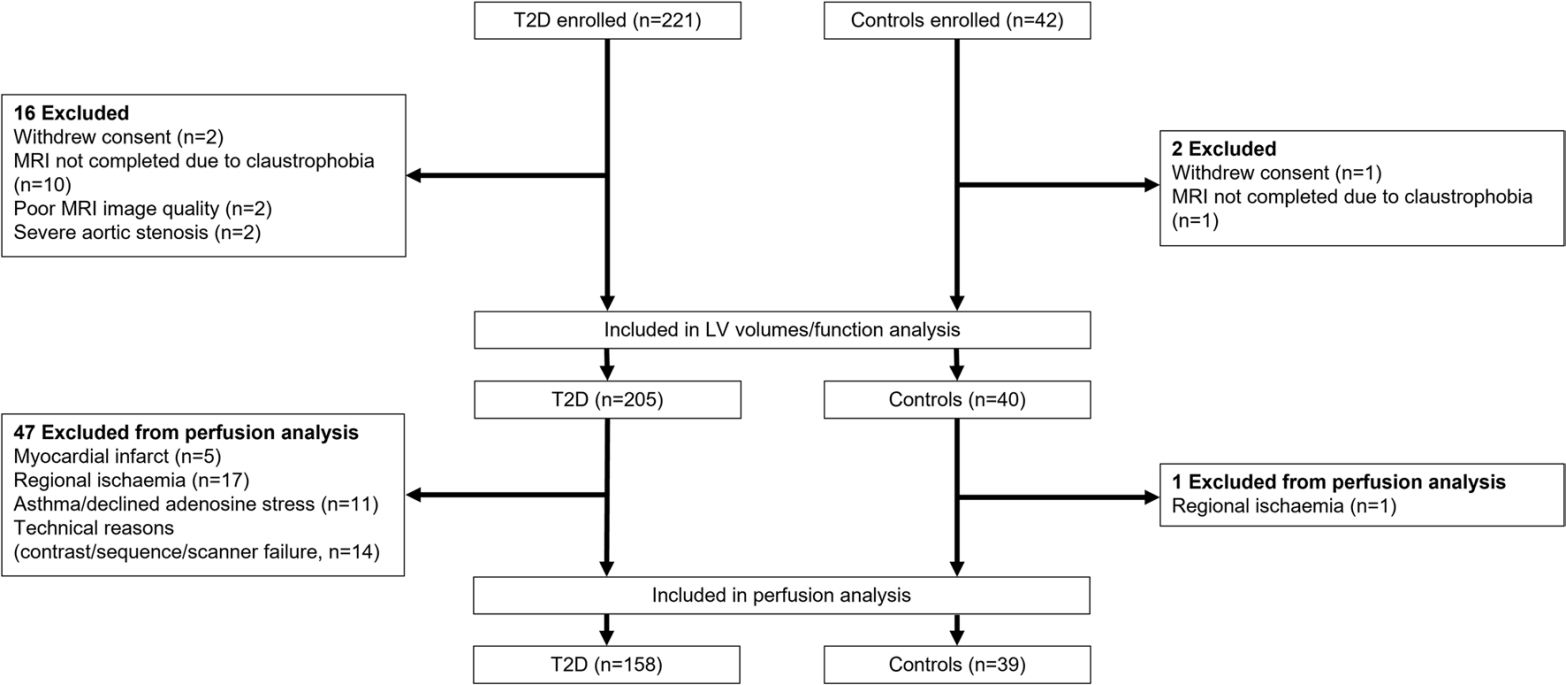
Study flowchart. LV indicates left ventricle; MRI: magnetic resonance imaging; T2D: type 2 diabetes

The study recruitment is summarized in Fig. 1. Two-hundred and twenty-one patients with T2D and forty-two controls were recruited. Sixteen patients with T2D and two controls were excluded, leaving two-hundred and five T2D and forty controls included for LV volumes and function analysis. For analysis of myocardial perfusion, participants found to have myocardial infarction (T2D n = 5) or regional ischemia (T2D n = 17, controls n = 1) on MRI were further excluded.

The baseline demographics and clinical characteristics of patients with T2D and controls are summarized in Table 1. Mean age of participants with T2D was 62.9 ± 7.0 years, 61% were males, and 72% were of white ethnicity. The control group was matched for age, sex, ethnicity, and smoking status. Those with T2D had a higher BMI than controls (31.0 ± 6.1 vs 26.6 ± 4.2 kg/m^2^, p < 0.001), and a higher proportion had hypertension and dyslipidemia. Anti-hypertensive and lipid-lowering medication use was higher in those with T2D. The proportion of those with T2D on single, two, and three or more anti-hypertensive medication were 27%, 22%, and 10%, respectively, while only 18% of controls were on a single anti-hypertensive medication.

**Table 1:** Baseline characteristics

	T2D (n = 205)	Controls (n = 40)	p value
Age, years	62.9 (7.0)	60.7 (7.7)	0.074
Sex, males	125 (61)	25 (63)	0.856
Ethnicity, n (%)			0.865
White	147 (72)	28 (70)	
Asian	48 (23)	11 (28)	
Other	10 (5)	1 (2)	
Smoking status, n (%)			0.138
Never smoked	114 (56)	23 (58)	
Ex-smoker	73 (36)	17 (42)	
Current smoker	18 (8)	0	
BMI, kg/m^2^	31.0 (6.1)	26.6 (4.2)	< 0.001
Duration of diabetes, years	9.0 (5.0–14.8)	–	–
Hypertension, n (%)	124 (60)	7 (18)	< 0.001
Hypercholesterolemia, n (%)	151 (74)	7 (18)	< 0.001
Medication, n (%)			
ACE inhibitor/ARB	98 (48)	4 (10)	0.001
Number of anti-hypertensives			< 0.001
0	83 (41)	33 (82)	
1	56 (27)	7 (18)	
2	46 (22)	0	
≥ 3	20 (10)	0	
Statin	142 (70)	8 (20)	< 0.001
Biguanide	151 (74)	–	–
Sulphonylurea	32 (16)	–	–
Thiazolidinediones	3 (2)	–	–
DPP-4 inhibitor	35 (17)	–	–
GLP-1 receptor agonist	21 (10)	–	–
SGLT-2 inhibitor	37 (18)	–	–
Insulin	35 (17)	–	–
Biochemistry			
Hemoglobin, g/dL	14.1 (1.5)	14.8 (1.2)	0.014
Estimated GFR, mL/min/1.73m^2^	84.6 (14.0)	84.9 (11.4)	0.913
Fasting glucose, mmol/L	8.1 (2.3)	5.2 (0.7)	< 0.001
HbA1c, mmol/mol	57.5 (13.3)	36.2 (3.9)	< 0.001
HbA1c, %	7.4 (1.2)	5.5 (0.4)	< 0.001
LDL, mmol/L	2.2 (0.8)	3.2 (0.9)	< 0.001
Cholesterol:HDL ratio	3.3 (0.9)	3.3 (1.1)	0.952
NT-proBNP, pg/mL	45 (24–79)	48 (29–99)	0.159
Albuminuria, n (%)	49 (24)	6 (15)	0.298

Values are presented as means (SD), median (interquartile range) or n (%). ACE indicates angiotensin-converting enzyme; ARB: angiotensin receptor blocker; BMI: body mass index; DPP-4: dipeptidylpeptidase-4; GFR: glomerular filtration rate; GLP-1: glucagon-like peptide-1; HbA1c: glycated hemoglobin; HDL: high-density lipoprotein; LDL: low-density lipoprotein; NT-proBNP: N-terminal pro-B-type natriuretic peptide; SGLT-2: sodium-glucose cotransporter-2

### Hemodynamics

There were no significant differences in office SBP and DBP between the groups (Table 2). Ambulatory SBP, but not DBP, measured over 24 h (128.8 ± 11.7 vs 123.0 ± 13.1 mmHg, p = 0.006), during daytime (133.1 ± 11.4 vs 128.4 ± 13.6 mmHg, p = 0.029) and night-time (119.2 ± 15.2 vs 112.4 ± 13.0 mmHg, p = 0.010) were significantly higher in patients with T2D compared to controls. There was no significant difference between the groups in the proportion of participants with a non-dipping pattern of BP. Only 11 participants with T2D had a reverse dipping pattern of blood pressure. As this equates to only 5% of the T2D cohort, further subgroup analysis of those with reverse dipping was not conducted. There was a high proportion of successful readings in those with T2D (median 86% daytime and 93% night-time, Additional file 1: Table S1). Both office (76.6 ± 12.6 vs 63.6 ± 8.8 bpm, p < 0.001) and ambulatory mean heart rate (76.8 ± 9.2 vs 67.8 ± 6.5 bpm, p < 0.001) measurements were significantly higher in patients with T2D compared to controls.

**Table 2:** Office and ambulatory hemodynamic results

	T2D (n = 205)	Controls (n = 40)	p value
Office			
SBP, mmHg	137.6 (17.5)	138.5 (21.1)	0.759
DBP, mmHg	82.3 (9.2)	85.0 (9.6)	0.089
Pulse pressure, mmHg	55.3 (14.1)	53.5 (15.7)	0.476
Heart rate, bpm	76.6 (12.6)	63.6 (8.8)	< 0.001
Ambulatory 24-h			
SBP, mmHg	128.8 (11.7)	123.0 (13.1)	0.006
DBP, mmHg	74.2 (7.2)	75.0 (7.8)	0.539
Pulse pressure, mmHg	54.6 (10.1)	48.0 (8.5)	< 0.001
Heart rate, bpm	76.8 (9.2)	67.8 (6.5)	< 0.001
Ambulatory daytime			
SBP, mmHg	133.1 (11.4)	128.4 (13.6)	0.029
DBP, mmHg	77.7 (7.2)	79.5 (7.9)	0.161
Pulse pressure, mmHg	55.3 (10.4)	48.9 (9.0)	< 0.001
Heart rate, bpm	79.8 (9.7)	70.2 (6.6)	< 0.001
Ambulatory night-time			
SBP, mmHg	119.2 (15.2)	112.4 (13.0)	0.010
DBP, mmHg	66.8 (9.2)	66.1 (8.6)	0.680
Pulse pressure, mmHg	52.5 (10.9)	46.3 (7.9)	0.001
Heart rate, bpm	70.6 (9.2)	62.8 (7.5)	< 0.001
Non-dipping pattern, n (%)	86 (42)	14 (35)	0.288

Values are presented as means (SD) or n (%). DBP indicates diastolic blood pressure; SBP: systolic blood pressure

### MRI and echocardiographic measures

**Fig. 2:**
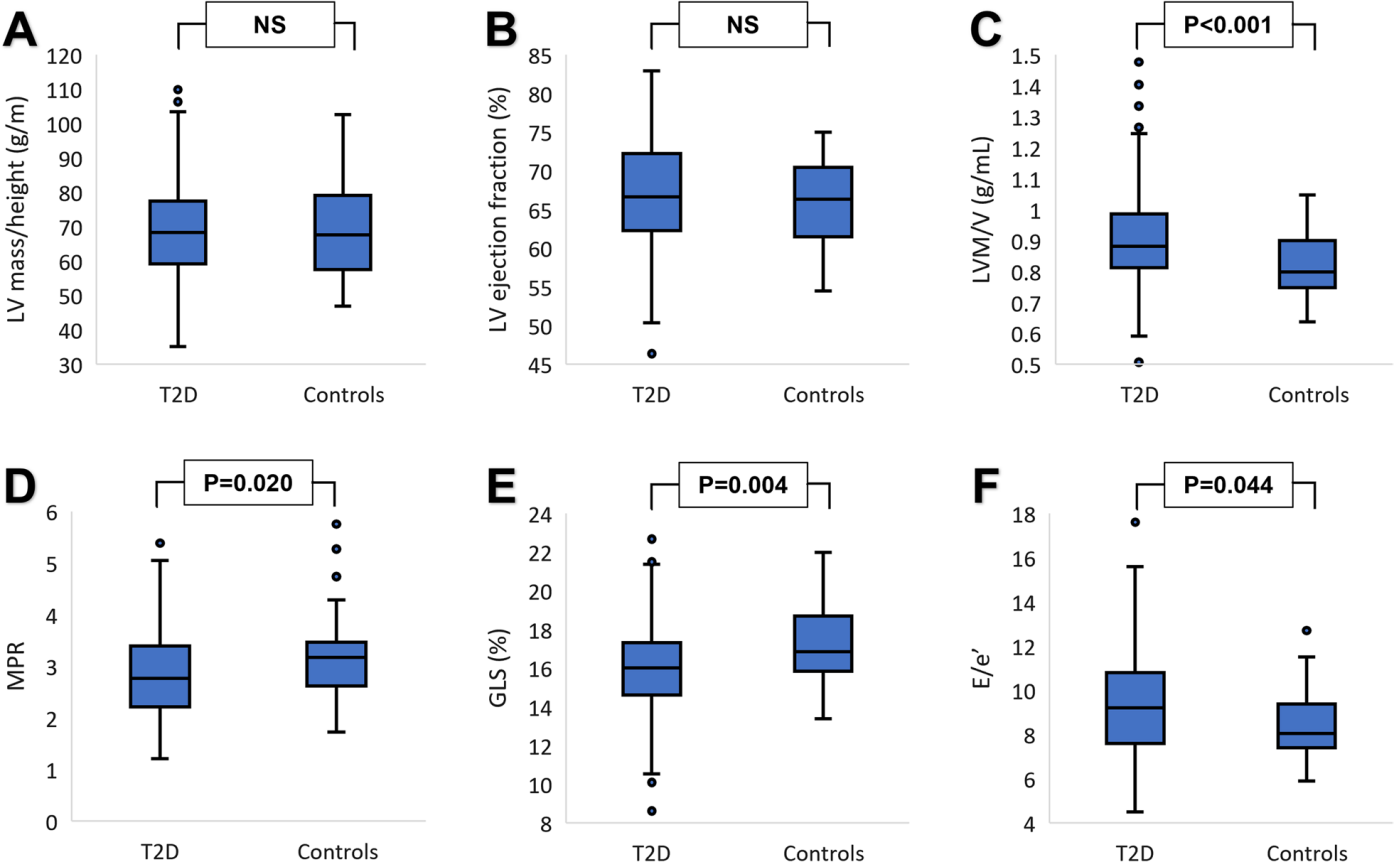
Left ventricular (LV) mass/height ratio (**A**), LV ejection fraction (**B**), LV mass/volume ratio (LVM/V) (**C**), myocardial perfusion reserve (MPR) (**D**), global longitudinal strain (GLS) (**E**), and diastolic function (E/e') (**F**) in type 2 diabetes (T2D) compared to controls. NS indicates not significant

**Fig. 3:**
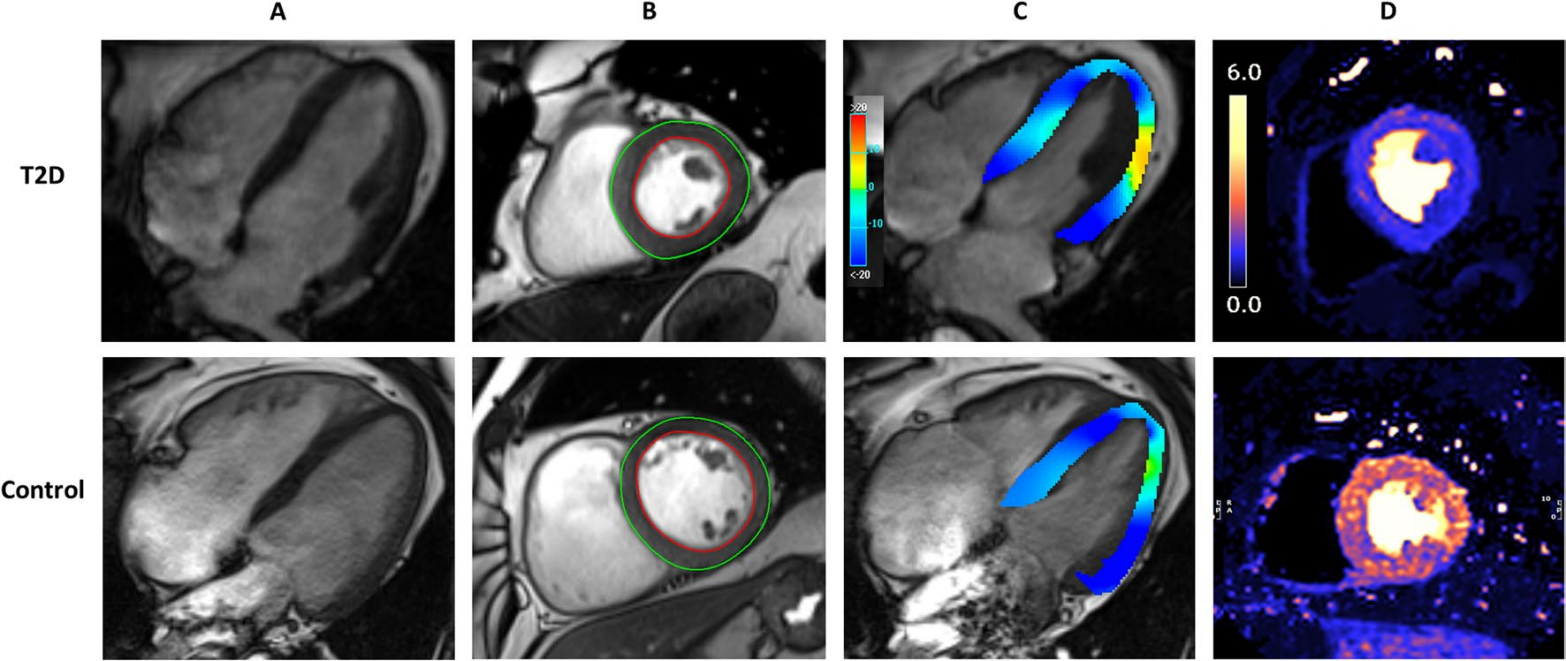
MRI images displaying the 4-chamber view (**A**) and mid- ventricular short-axis slice (**B**) during diastole, global longitudinal strain assessment (GLS) (**C**), and stress myocardial perfusion map in mid-ventricular short-axis slice (**D**). Top row images were from a 59-year-old male with type 2 diabetes (T2D), BMI of 40 kg/m^2^; left ventricular mass (LVM) 146 g, left ventricular end-diastolic volume (LVEDV) 122 mL, left ventricular mass to volume ratio (LVM/V) 1.2 g/mL, GLS 13.0%, stress myocardial blood flow (MBF) 1.2 mL/min/g, rest MBF 0.6 mL/min/g, and myocardial perfusion reserve (MPR) 2.0. The bottom row images were from a 64-year-old male non-diabetic control, BMI of 32 kg/m^2^; LVM 179 g, LVEDV 223 mL, LVM/V 0.8 g/mL, GLS 15.3%, stress MBF 2.2 mL/min/g, rest MBF 0.6 mL/min/g, and MPR 3.7. Note for GLS with darker blue indicates greater shortening and higher strain

Imaging data are shown in Fig. 2 and Table 3. Participants with T2D had lower absolute LV end-diastolic volume (132 ± 34 vs 148 ± 33 mL, p < 0.001) and left atrial volume (61.9 ± 19.3 vs 69.6 ± 25.0 mL, p = 0.033) but similar absolute LV mass (117 ± 27 vs 121 ± 31 g, p = 0.409). People with T2D had increased LV concentric remodeling, demonstrated by a higher LVM/V ratio (0.91 ± 0.15 vs 0.82 ± 0.11 g/mL, p < 0.001). Systolic function measured by LV ejection fraction was similar in both groups, but GLS was lower in T2D than controls (16.0 ± 2.3 vs 17.2 ± 2.1%, p = 0.004). Diastolic function was worse in T2D compared to controls as evidenced by a lower E/A ratio (0.87 ± 0.18 vs 1.00 ± 0.25, p < 0.001) and higher E/e’ (9.30 ± 2.43 vs 8.47 ± 1.53, p = 0.044). Resting myocardial blood flow was similar between the groups, but stress myocardial blood flow (1.78 ± 0.55 vs 2.00 ± 0.63 mL/min/g, p = 0.032) and MPR (2.82 ± 0.83 vs 3.18 ± 0.82, p = 0.020) were lower in people with T2D. ECV was higher in those with T2D (27.0 ± 2.7 vs 25.6 ± 1.7%, p = 0.003). Representative MRI image examples of T2D and control are illustrated in Fig. 3. The intra-observer and inter-observer variability for echocardiographic measurements were excellent with all ICC’s > 0.90 (Additional file 1: Table S2).

**Table 3:** Echocardiographic and MRI measures

	T2D (n = 205)	Controls (n = 40)	p value
Echocardiography measures			
E/A ratio	0.87 (0.18)	1.00 (0.25)	< 0.001
E/e’ ratio	9.30 (2.43)	8.47 (1.53)	0.044
MRI measures			
LVM/height, g/m	68.9 (14.0)	69.8 (15.0)	0.718
LVEDV/height, mL/m	77.5 (17.6)	85.7 (15.7)	0.007
LV mass/volume, g/mL	0.91 (0.15)	0.82 (0.11)	< 0.001
LV ejection fraction, %	66.9 (7.3)	65.7 (6.4)	0.351
Global Longitudinal Strain, %	16.0 (2.3)	17.2 (2.1)	0.004
Stress MBF, mL/min/g†	1.78 (0.55)	2.00 (0.63)	0.032
Rest MBF, mL/min/g†	0.65 (0.18)	0.64 (0.15)	0.682
Myocardial Perfusion Reserve†	2.82 (0.83)	3.18 (0.82)	0.020
Native T1, ms	1227 (37)	1210 (33)	0.008
Extra Cellular Volume, %	27.0 (2.7)	25.6 (1.7)	0.003
LA maximal volume, mL	61.9 (19.3)	69.6 (25.0)	0.033
Mean aortic distensibility, 10^–3^ mmHg^−1^	6.28 (2.96)	6.67 (2.93)	0.452

Values are presented as means (SD). BSA indicates body surface area; E/A: early to late diastolic mitral inflow velocity ratio; E/e’: early diastolic mitral inflow to annular velocity ratio; LA: left atrium; LVEDV: left ventricular end-diastolic volume; LVM: left ventricular mass; MBF: myocardial blood flow

^†^Myocardial blood flow analysis performed after excluding those with infarct (T2D n = 5, controls n = 0), regional ischemia (T2D n = 17, controls n = 1), or did not undergo adenosine stress due to technical/clinical reasons (T2D n = 25)

### Association between office and ambulatory BP with left ventricular remodeling, perfusion reserve, systolic and diastolic function in T2D

The univariable correlations of BP and measures of cardiac structure and function in T2D are displayed in Additional file 1: Table S3. Office SBP was inversely correlated with MPR and positively correlated with E/e’. Ambulatory SBP (24-h, daytime, and night-time) were positively correlated with LVM/V and E/e’, and inversely correlated with MPR. Office DBP was inversely correlated with GLS and ECV. Ambulatory DBP was inversely correlated with GLS, E/e’ (24 h and daytime DBP) and ECV (daytime DBP only).

**Fig. 4:**
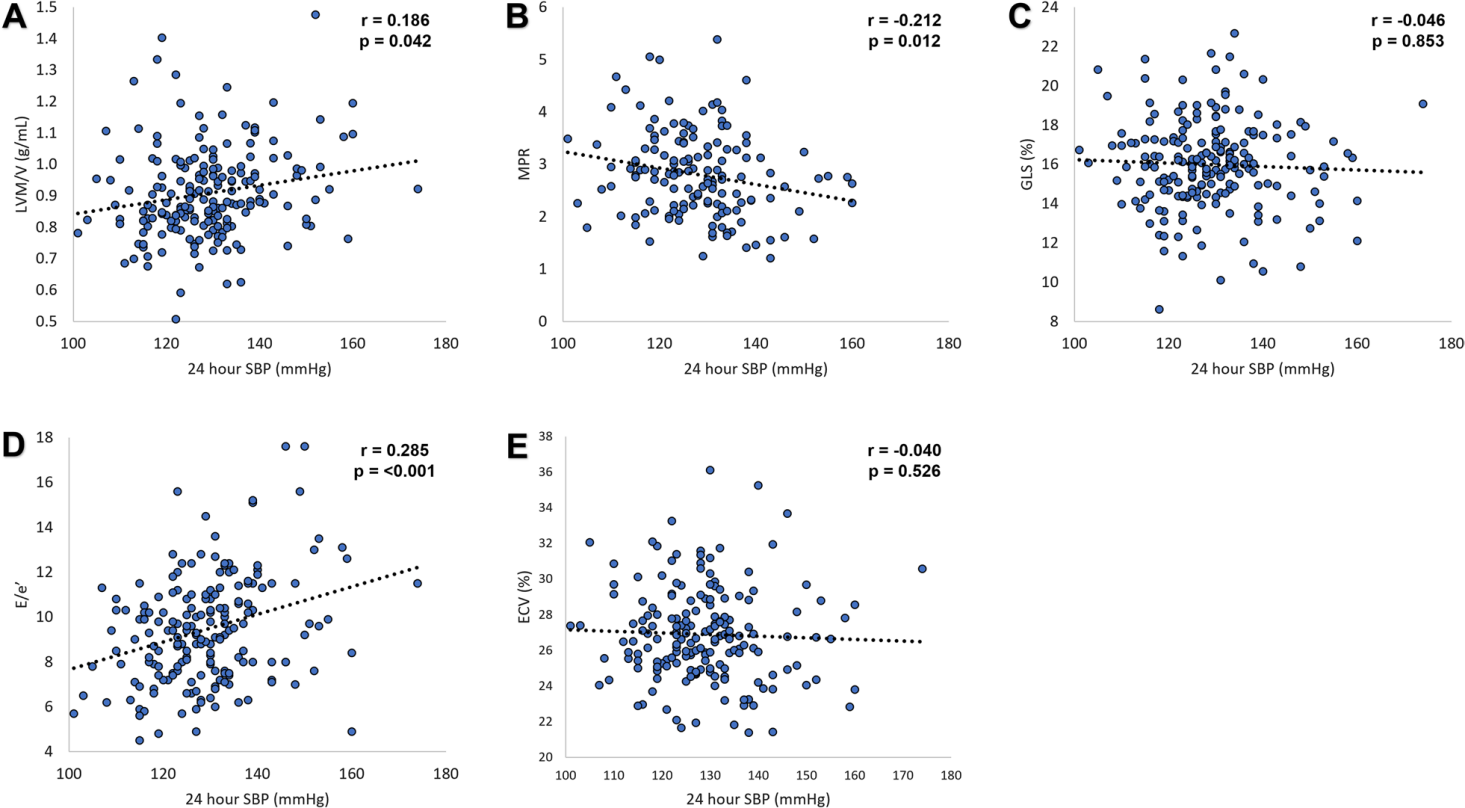
Correlation between ambulatory 24-h systolic blood pressure (SBP) with left ventricular mass/volume ratio (LVM/V) (**A**), myocardial perfusion reserve (MPR) (**B**), global longitudinal strain (GLS) (**C**), diastolic function (E/e’) (**D**), and myocardial extracellular volume fraction (ECV) (**E**) in people with type 2 diabetes. p values displayed are adjusted for age, male sex, white ethnicity, never smoker, body mass index, HbA1c, number of anti-hypertensives, insulin, sodium-glucose co-transporter-2 inhibitor, and glucagon-like peptide-1 receptor use

Results for multivariable linear regression analyses adjusting for clinical variables are displayed in Table 4. Following adjustments, office SBP remained inversely associated with only MPR. Ambulatory SBP remained positively associated with LVM/V (24-h) and E/e’ (24-h, daytime, and night-time), and inversely associated with MPR (24-h,daytime, and night-time). Office DBP remained inversely associated with only GLS, while it emerged ambulatory DBP was positively associated with LVM/V (24-h). Figure 4 displays the linear correlation scatterplots for 24-h SBP with each imaging measure. There was no statistically significant interaction between sex and blood pressure variables for each of the key imaging outcome measures (all p > 0.05, results not shown).

**Table 4:** Multivariable association of blood pressure with MRI and echocardiographic parameters in T2D

	LVM/V			MPR			GLS			E/e’			ECV		
	B (SE)	β	p value	B (SE)	β	p value	B (SE)	β	p value	B (SE)	β	p value	B (SE)	β	p value
Office SBP	0.001 (0.001)	0.113	0.144	**−** **0.008** **(0.004)**	**−** **0.181**	**0.048**	**−** 0.003 (0.010)	**−** 0.020	0.805	0.018 (0.010)	0.125	0.089	**−** 0.008 (0.011)	**−** 0.053	0.461
24-h SBP	**0.002** **(0.001)**	**0.165**	**0.031**	**−** **0.016 (0.007)**	**−** **0.218**	**0.016**	**−** 0.003 (0.015)	**−** 0.016	0.836	**0.058 (0.015)**	**0.273**	** < 0.001**	**−** 0.011 (0.016)	**−** 0.046	0.515
Daytime SBP	0.002 (0.001)	0.147	0.051	**−** **0.018 (0.007)**	**−** **0.227**	**0.011**	0.006 (0.015)	0.028	0.714	**0.059 (0.015)**	**0.268**	** < 0.001**	**−** 0.018 (0.017)	**−** 0.074	0.291
Night-time SBP	0.001 (0.001)	0.128	0.098	**−** **0.013 (0.006)**	**−** **0.192**	**0.039**	**−** 0.015 (0.012)	**−** 0.099	0.209	**0.031 (0.012)**	**0.187**	**0.011**	0.000 (0.013)	**−** 0.032	0.975
Office DBP	0.002 (0.001)	0.105	0.164	**−** 0.002 (0.008)	**−** 0.028	0.758	**−** **0.049 (0.019)**	**−** **0.200**	**0.008**	-0.002 (0.019)	**−** 0.008	0.913	**−** 0.015 (0.021)	**−** 0.050	0.476
24-h DBP	**0.004** **(0.002)**	**0.201**	**0.016**	**−** 0.014 (0.011)	**−** 0.124	0.206	**−** 0.047 (0.027)	**−** 0.150	0.078	0.005 (0.028)	0.016	0.842	**−** 0.016 (0.029)	**−** 0.044	0.573
Daytime DBP	0.003 (0.002)	0.166	0.050	**−** 0.014 (0.012)	**−** 0.122	0.222	**−** 0.039 (0.027)	**−** 0.123	0.157	0.005 (0.028)	0.016	0.848	**−** 0.038 (0.030)	**−** 0.100	0.205
Night-time DBP	0.002 (0.001)	0.147	0.061	**−** 0.013 (0.010)	**−** 0.131	0.176	**−** 0.034 (0.020)	**−** 0.140	0.081	0.007 (0.020)	0.027	0.721	0.009 (0.021)	0.030	0.681

Values are presented as unstandardised coefficient (B), standard error (SE), and standardised coefficient (β). Bold indicates statistically significant coefficient

P values adjusted for age, male sex, white ethnicity, never smoked, body mass index, diabetes duration, HbA1c, estimated glomerular filtration rate, albuminuria, NT-proBNP, number of anti-hypertensive(s), insulin, sodium-glucose co-transporter-2 inhibitor, and glucagon-like peptide-1 receptor agonist use

DBP indicates diastolic blood pressure; ECV: extracellular volume fraction; E/e’: early diastolic mitral inflow velocity to mitral annular velocity; LVM/V: left ventricular mass to volume ratio; MPR: myocardial perfusion reserve; SBP: systolic blood pressure

## Discussion

This is the first study to demonstrate associations between ambulatory BP and MRI markers of subclinical cardiac dysfunction in people with T2D. We have shown that in a well-phenotyped cohort of T2D without CVD or symptoms, ambulatory SBP is associated with LV concentric remodeling, microvascular dysfunction, and diastolic impairment, independent of other major clinical risk factors. These associations remained significant despite adjusting for important prognostic biomarkers such as NT-proBNP [28] and albuminuria [29]. Additionally, ambulatory DBP was independently associated with increased LV concentric remodeling. Office BP was not significantly different between T2D and controls. Among people with T2D, office SBP was associated with MPR only, whilst office DBP with GLS.

Our results are consistent with previous findings of LV concentric remodeling seen in cohorts with hypertension and/or T2D [30–32]. Myocardial remodeling in systemic hypertension and diabetes share common pathophysiological features including cardiomyocyte hypertrophy and myocardial interstitial fibrosis [33, 34]. These changes are secondary to the combined effects of mechanical stress from increased afterload in hypertension and the pro-inflammatory hyperglycemic state in T2D, resulting in increased collagen production. In our case–control comparison, we have confirmed that those with T2D had higher ECV fraction, a surrogate marker for diffuse interstitial fibrosis. However, ECV was not associated with BP in our multivariable model, suggesting that other factors, such as inflammation, may be driving the increase in ECV in T2D. Another important finding is that ambulatory DBP was also independently associated with myocardial remodeling, highlighting the importance of achieving both systolic and diastolic BP targets in cardiovascular risk management among T2D. Furthermore, a previous meta-analysis found that the most significant regression of LV hypertrophy in hypertensive patients is seen in those who had DBP reduction of ≥ 10 mmHg [35].

Diabetes and hypertension act synergistically in the pathogenesis of microvascular dysfunction through various mechanisms including endothelial dysfunction, capillary rarefaction, and arteriolar smooth muscle hypertrophy [36]. In the absence of obstructive epicardial coronary disease, MPR is a measure of microvascular function. In the current study, those with T2D had lower stress MBF compared to controls, leading to a lower MPR. Both ambulatory and office SBP were inversely associated with MPR following multivariable adjustment. Our results are additive to previous findings that hypertension has the strongest correlation with MPR over other risk factors such as diabetes and dyslipidemia [37]. Importantly, a lower MPR is associated with impaired myocardial contractile function [38], impaired exercise capacity [17], and prognostic of adverse cardiovascular outcomes [39, 40].

Our results showing impairment of systolic (lower GLS) and diastolic function (higher E/e’) among T2D are corroborated by numerous studies [41, 42]. Apart from stiffening due to interstitial fibrosis and myocardial hypertrophy mentioned above, other accepted mechanisms contributing to subclinical LV dysfunction include perturbations at a cellular level such as impaired myocardial energy metabolism and calcium handling [34]. In the present study, only office DBP remained inversely associated with GLS following multivariable adjustments. However, this isolated finding cannot be easily explained, especially in the absence of a significant association between GLS and ambulatory blood pressure. The positive association between E/e’, an indicator of increased LV filling pressure, with ambulatory SBP, independent of other risk factors, is in keeping with the continuum between hypertension, concentric remodeling, and diastolic dysfunction. This suggests that a lower SBP may have a greater impact on improving diastolic function in asymptomatic people with T2D.

Our results are consistent with a previous echocardiographic study (n = 577, mean age 70.2 years, 40% male) that found direct independent associations between LV mass with ambulatory SBP and DBP, and between E/e’ with ambulatory SBP [43]. However, they also found that GLS was associated with ambulatory SBP and DBP, in contrast to our results. Several other studies have also shown associations between increased ambulatory DBP with impaired GLS in hypertensive subjects, regardless of presence of LV hypertrophy [44, 45]. However, there are key differences between our studies which may explain the discrepancy. Firstly, these studies were conducted in predominantly hypertensive cohorts which have a different risk factor profile from a diabetes cohort. Secondly, they measured GLS using echocardiography, which is highly dependent on image quality, a particular challenge in obese individuals, and not directly comparable to MRI [46]. Kim et al. also noted that the association between ambulatory DBP and GLS was stronger in those without LV hypertrophy than those with hypertrophy [45]. We speculate that, once LV remodeling develops, the association between ambulatory BP and GLS is attenuated, as seen in our cohort.

Elevated sleep-time blood pressure has been shown to be a stronger predictor of CVD risk, in particular for heart failure, compared to awake or 24-h mean blood pressure [47]. In our study, we found that higher night-time systolic blood pressure was independently associated with diastolic and microvascular dysfunction, and showed a non-significant trend towards LV concentric remodeling.

Whilst a causal relationship cannot be determined from this observational study, there may be a role of more aggressive blood pressure control, especially in younger or middle-aged individuals who have the greatest lifetime risk of developing heart failure and cardiovascular complications [48] and who are less prone to side effects such as postural hypotension compared to older patients. Randomized controlled trials of sodium-glucose co-transporter 2 inhibitors and glucagon-like peptide-1 receptor agonists, which have diuretic and BP lowering effects, have shown promising results in reducing cardiovascular mortality and/or heart failure hospitalizations in cohorts with T2D or heart failure [49–52]. These agents also reduce LV mass [53] but it is unclear how much of this effect is due to blood pressure lowering alone and whether MPR is also improved. Future studies should assess the degree of reverse remodeling with these newer glucose lowering agents versus or combined with tight blood pressure reduction.

### Strengths and limitations

Our study has several strengths, including detailed phenotyping using standardized state-of-the-art MRI imaging techniques to assess the link between coronary microvascular function and markers of diffuse myocardial fibrosis with blood pressure. We have prospectively recruited a multi-ethnic cohort of T2D participants without cardiovascular symptoms or diagnosis, which is a representative sample population who are at risk of early heart failure, and where intervention may have the greatest impact on modifying outcomes. However, a few limitations should be considered. Firstly, our sample size is relatively small but given the comprehensive phenotyping with contrast-enhanced cardiac MRI, our study is one of the largest to assess effects of BP on cardiac structure and function in people with T2D. Second, our control group of people without a history of T2D is, however, not a purely healthy cohort. The controls included people with increased BMI, hypertension, and hypercholesterolemia, selected to reflect a representative sample of our regional population without T2D. Third, we did not have invasive coronary imaging which is the modality of choice to assess coronary patency. However, we excluded patients with regional ischemia on MRI perfusion imaging which has excellent diagnostic accuracy to detect obstructive coronary disease [20, 21]. Fourth, the cross-sectional, observational nature of this study cannot determine a causal relationship or any direction of causality. Fifth, we performed cardiac MRI scans exclusively at 3 Tesla field strength. Further confirmation of these results on a 1.5 Tesla platform is warranted, given the more widespread use of this MRI field strength and potential inter-field variability. Lastly, we acknowledge our ambulatory BP is measured over 24 h rather than 48 h, which has been shown to have higher reproducibility and reliable classification of dipping status [54].

## Conclusions

In this study, we sought to determine the relationship between BP and imaging measures of cardiac structure and function in asymptomatic individuals with T2D. We found that ambulatory SBP is independently associated with LV concentric remodeling, MPR, and diastolic function, whilst office SBP is only independently associated with MPR.

## Supplementary Information

**Additional file 1: Table S1.** Percentages of successful ambulatory blood pressure recordings. **Table S2.** Intra- and interobserver variability for E/A and E/e'. **Table S3.** Univariable correlation of blood pressure with MRI and echocardiographic parameters in T2D.

## Data Availability

The datasets used and/or analyzed during the current study are available from the corresponding author on reasonable request.

## Abbreviations

BP: Blood pressure
BMI: Body mass index
CVD: Cardiovascular disease
DBP: Diastolic blood pressure
E/e’: Early diastolic mitral inflow velocity to early diastolic mitral annular velocity ratio
ECV: Extracellular volume fraction
GLS: Global longitudinal strain
HbA1c: Glycosylated hemoglobin
LGE: Late gadolinium enhancement
LV: Left ventricle
LVM/V: Left ventricular mass to end-diastolic volume ratio
MPR: Myocardial perfusion reserve
MRI: Magnetic resonance imaging
SBP: Systolic blood pressure
T2D: Type 2 diabetes
