# Supplementary material for: Association of ambulatory blood pressure with coronary microvascular and cardiac dysfunction in asymptomatic type 2 diabetes
Source: Cardiovasc Diabetol. 2022 May 28;21:85. doi: 10.1186/s12933-022-01528-2 (PMC9148453; doi:10.1186/s12933-022-01528-2)
Supplement: Supplementary file 1 — Additional file 1: Table S1. Percentages of successful ambulatory blood pressure recordings. Table S2. Intra- and interobserver variability for E/A and E/e'. Table S3. Univariable correlation of blood pressure with MRI and echocardiographic parameters in T2D. [file 12933_2022_1528_MOESM1_ESM.docx]

# Supplementary

Table S1 Percentages of successful ambulatory blood pressure recordings

|  | Successful ambulatory blood pressure recordings (%) | |
| --- | --- | --- |
|  | T2D | Controls |
| 24-hour | 89 (80 - 93) | 93 (86 - 98) |
| Daytime | 86 (75 - 91) | 93 (83 - 97) |
| Night-time | 100 (94 - 100) | 100 (100 - 100) |

Values are presented as medians (interquartile range).

Table S2 Intra- and interobserver variability for E/A and E/e'

| Observer variability | Intraclass correlation coefficient (95% CI) | |
| --- | --- | --- |
|  | E/A | E/e’ |
| Intra-observer (observer 1 vs observer 1) | 0.986 (0.948 - 0.996) | 0.979 (0.921 - 0.995) |
| Inter-observer (observer 1 vs observer 2) | 0.984 (0.937 – 0.996) | 0.912 (0.699 - 0.977) |

Table S3 Univariable correlation of blood pressure with MRI and echocardiographic parameters in T2D

|  | LVM/V | | MPR | | GLS | | E/e’ | | ECV | |
| --- | --- | --- | --- | --- | --- | --- | --- | --- | --- | --- |
|  | r | p value | r | p value | r | p value | r | p value | r | p value |
| Office SBP | 0.107 | 0.126 | **-0.210** | **0.009** | -0.018 | 0.796 | **0.190** | **0.007** | -0.087 | 0.229 |
| 24-hour SBP | **0.186** | **0.009** | **-0.212** | **0.011** | -0.046 | 0.527 | **0.285** | **<0.001** | -0.040 | 0.597 |
| Daytime SBP | **0.175** | **0.015** | **-0.225** | **0.007** | -0.014 | 0.847 | **0.275** | **<0.001** | -0.071 | 0.342 |
| Night-time SBP | **0.151** | **0.039** | **-0.179** | **0.034** | -0.115 | 0.119 | **0.200** | **0.007** | -0.014 | 0.851 |
| Office DBP | 0.065 | 0.354 | -0.044 | 0.592 | **-0.216** | **0.002** | -0.061 | 0.391 | **-0.150** | **0.038** |
| 24-hour DBP | 0.125 | 0.083 | -0.004 | 0.958 | **-0.209** | **0.003** | **-0.172** | **0.018** | -0.107 | 0.152 |
| Daytime DBP | 0.091 | 0.207 | -0.002 | 0.985 | **-0.191** | **0.008** | **-0.191** | **0.008** | **-0.153** | **0.041** |
| Night-time DBP | 0.107 | 0.143 | 0.010 | 0.907 | **-0.190** | **0.009** | -0.109 | 0.142 | -0.030 | 0.691 |

Bold indicates statistically significant correlation coefficient.

DBP indicates diastolic blood pressure; ECV, extracellular volume fraction; E/e’, early diastolic mitral inflow velocity to mitral annular velocity; LVM/V, left ventricular mass to volume ratio; MPR, myocardial perfusion reserve; SBP, systolic blood pressure.
